# Supplementary material for: Development of Machine Learning Models to Predict Platinum Sensitivity of High-Grade Serous Ovarian Carcinoma
Source: Cancers (Basel). 2021 Apr 14;13(8):1875. doi: 10.3390/cancers13081875 (PMC8070756; doi:10.3390/cancers13081875)
Supplement: Supplementary file 1 [file cancers-13-01875-s001.pdf]

Supplementary Materials

Development of Machine Learning Models to Predict Platinum Sensitivity of High-Grade Serous Ovarian Carcinoma

Suhyun Hwangbo, Se Ik Kim, Ju-Hyun Kim, Kyung Jin Eoh, Chanhee Lee, Young Tae Kim, Dae-Shik Suh, Taesung Park and Yong Sang Song

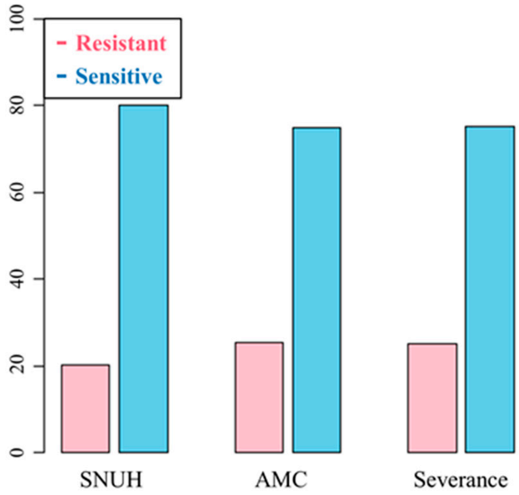

Figure S1. Distribution of platinum-sensitive and -resistant patients by each institution.

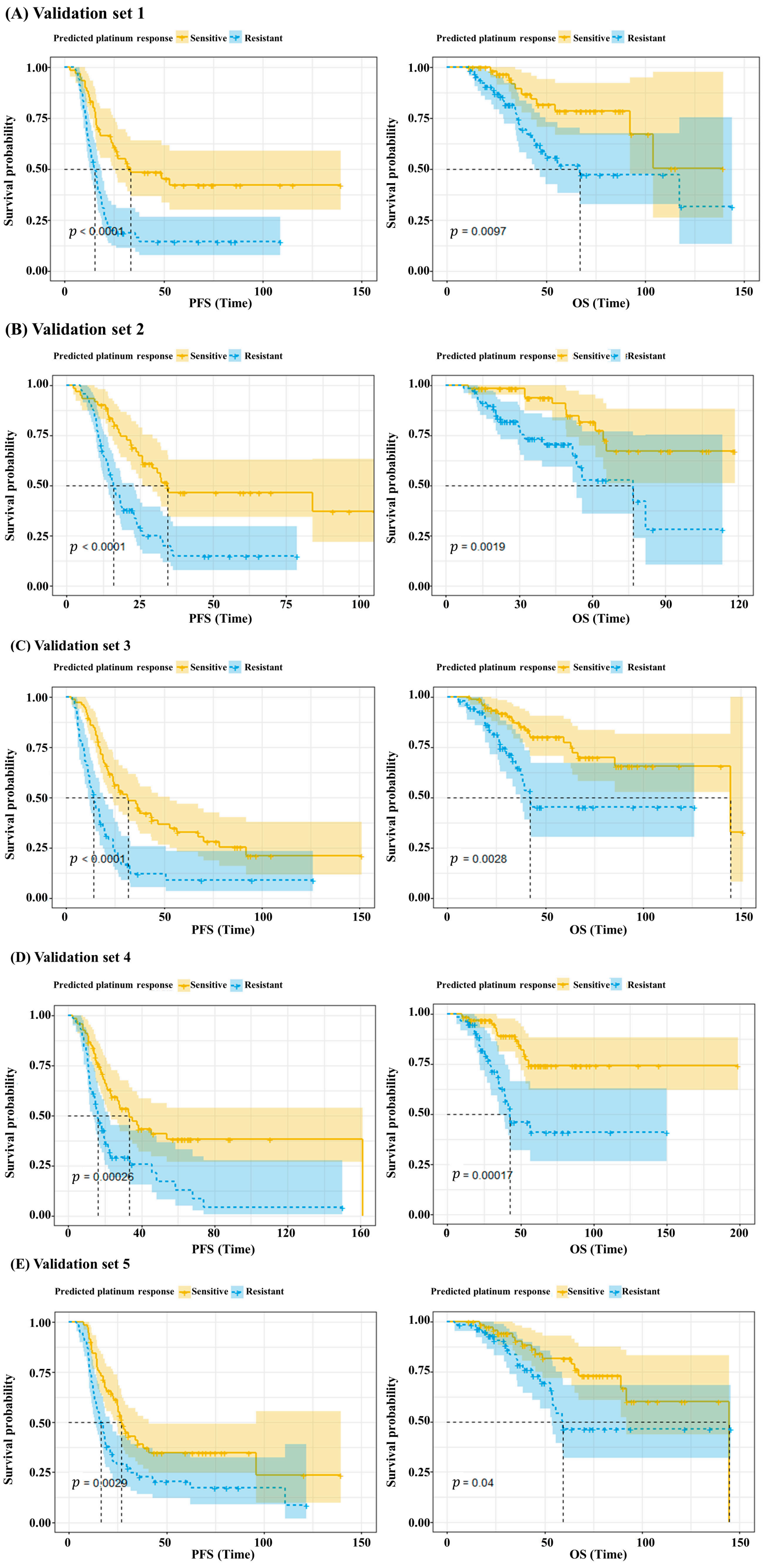

Figure S2. Comparisons of survival outcomes between the platinum-sensitive and -resistant groups predicted by the six-variable, LR model using the cut-off value of 0.175 in each validation set. Left, progress-free survival; Right, overall survival. (**A**–**E**) represent the results for the five validation sets.

Table S1. Independent variables used in the analysis.

Domain	No. of Variables	List
Basic information	3	Age, Parity, Menopausal state
Co-morbidities	3	Hypertension, Diabetes, Dyslipidemia
Personal and family history of cancers	5	Personal history of breast cancer, Familial history of breast cancer *, No. of family members with breast cancer*, Family history of gynecologic cancer *, No. of family members with gynecologic cancer*
Biometry	3	Height, Body weight, BMI
Tumor marker	1	Serum CA-125 levels at diagnosis
Laboratory tests	1	Hemoglobin
Staging and pathology	5	Origin, FIGO stage, Pelvic LN status, Para-aortic LN status, Pleural effusion
Neoadjuvant chemotherapy	2	NAC, Cycle of NAC
Surgical findings and procedure	17	LN dissection, No. of harvested LNs, No. of positive LNs, Large bowel resection, Upper abdominal surgery, Involvement of pelvic tissue other than uterus and tube, Involvement of bladder or rectal mucosa, Involvement of small bowel and mesentery, Involvement of colon other than rectosigmoid, Involvement of liver surface, Involvement of diaphragm, Involvement of spleen, Liver parenchyma metastasis, Involvement of other abdominal tissue, Lung metastasis, Supraclavicular LN metastasis, Residual tumor size after PDS/IDS
Frontline chemotherapy	2	Total cycles of frontline chemotherapy, Frontline chemotherapy regimen

Detailed information on the type of collected variables was the same as our previous study [1] except for differential blood cell counts at initial diagnosis, which were not collected in this study. Abbreviations: BMI, body mass index; CA-125, cancer antigen 125; FIGO, International Federation of Gynecology and Obstetrics; IDS, interval debulking surgery; LN, lymph node; NAC, neoadjuvant chemotherapy; PDS, primary debulking surgery. * Up to 2nd degree. Reference: 1. Kim, S.I.; Song, M.; Hwangbo, S.; Lee, S.; Cho, U.; Kim J.H.; Lee, M.; Kim, H.S.; Chung, H.H.; Suh, D.S.; et al. Development of Web-Based Nomograms to Predict Treatment Response and Prognosis of Epithelial Ovarian Cancer. Cancer Res. Treat. **2019**, *51*, 1144–1155.

Table S2. List of machine learning methods used in this study.

Method	Package	Version
Random forest	randomForest	4.6–14
Support Vector Machine	e1071	1.7–4
	keras	2.4.3
Deep Neural Network	scikit-learn	0.23.1
	tensorflow	2.3.0

Table S3. Surgical procedures and findings.

Characteristics	Missing Rate (%)	All (*n* = 1002, %)	Platinum-Sensitive (*n* = 779, %)	Platinum-Resistant (*n* = 223, %)	*p*
**Surgical procedures**					
LN dissection	4.3	835 (87.1)	652 (88.1)	183 (83.6)	0.100
No. of harvested LNs	1.0	27.5 ± 20	28 ± 21	25.8 ± 19	0.138
No. of positive LNs	12.8	6.2 ± 11	5.7 ± 10	8.4 ± 11	0.003
Large bowel resection	6.2	320 (34)	238 (32.7)	82 (38.7)	0.124
Upper abdominal surgery	6.8	291 (31.2)	215 (29.7)	76 (36.4)	0.078
**Surgical findings and tumor involvement**					
Pelvic LN metastasis	16.7	437 (52.3)	310 (47.5)	127 (69.8)	<0.001
Para-aortic LN metastasis	24.2	396 (52.1)	294 (49.2)	102 (63.0)	0.002
Pelvic tissue other than uterus and tube	20.5				0.102
No		224 (28.1)	186 (29.9)	38 (21.8)	
Microscopic		53 (6.6)	39 (6.3)	14 (8.0)	
Macroscopic		520 (65.2)	398 (63.9)	122 (70.1)	
Bladder or rectal mucosa	18.9	95 (11.7)	70 (11.0)	25 (14.0)	0.345
Small bowel and mesentery	20.4				<0.001
No + Microscopic		481 (60.3)	405 (64.8)	76 (43.9)	
Macroscopic, ≤2 cm		264 (33.1)	180 (28.8)	84 (48.6)	
Macroscopic, >2 cm		53 (6.6)	40 (6.4)	13 (7.5)	
Colon other than rectosigmoid	20.1				0.038
No + Microscopic		509 (63.5)	413 (65.8)	96 (55.5)	
Macroscopic, ≤2 cm		159 (19.9)	115 (18.3)	44 (25.4)	
Macroscopic, >2 cm		133 (16.6)	100 (15.9)	33 (19.1)	
Diaphragm	19.9				<0.001
No + Microscopic		467 (58.2)	384 (61.3)	83 (46.9)	
Macroscopic, ≤2 cm		264 (32.9)	184 (29.4)	80 (45.2)	
Macroscopic, >2 cm		72 (9.0)	58 (9.3)	14 (7.9)	
Liver surface	20.2				0.051
No + Microscopic		630 (78.8)	504 (80.5)	126 (72.4)	
Macroscopic, ≤2 cm		127 (15.9)	93 (14.9)	34 (19.5)	
Macroscopic, >2 cm		43 (5.4)	29 (4.6)	14 (8.0)	
Liver parenchyma metastasis	5.1	40 (4.2)	25 (3.3)	15 (7.4)	0.019
Spleen capsule	19.5				0.063
No + Microscopic		720 (89.2)	570 (90.5)	150 (85.2)	
Macroscopic, ≤2 cm		46 (5.7)	30 (4.8)	16 (9.0)	
Macroscopic, >2 cm		41 (5.1)	30 (4.8)	11 (6.2)	
Spleen parenchyma metastasis	19.5	18 (2.2)	15 (2.4)	3 (1.7)	0.796
Other abdominal tissue	19.4				0.229
No + Microscopic		443 (54.8)	356 (56.4)	87 (49.2)	
Macroscopic, ≤2cm		263 (32.5)	198 (31.4)	65 (36.7)	
Macroscopic, >2cm		102 (12.6)	77 (12.2)	25 (14.1)	
Lung metastasis	5.2	19 (2.0)	16 (2.1)	3 (1.5)	0.760
Supraclavicular LN metastasis	1.9	74 (7.5)	51 (6.6)	23 (10.7)	0.065
Pleural effusion	5.7	107 (11.3)	70 (9.4)	37 (18.2)	<0.001

Data are presented as mean ± standard deviation for continuous or number (%) for categorical variables. Detailed information on the type of collected variables was the same as our previous study [1] except for differential blood cell counts at initial diagnosis, which were not collected in this study. Abbreviations: LN, lymph node. Reference: 1. Kim, S.I.; Song, M.; Hwangbo, S.; Lee, S.; Cho, U.; Kim J.H.; Lee, M.; Kim, H.S.; Chung, H.H.; Suh, D.S.; et al. Development of Web-Based Nomograms to Predict Treatment Response and Prognosis of Epithelial Ovarian Cancer. Cancer Res. Treat. **2019**, *51*, 1144–1155.

Table S4. Fitted results of the logistic regression model used for nomogram development.

Variable	Estimate	SE	*p*
(Intercept)	−5.693	0.820	<0.001
Age	0.031	0.010	0.003
Serum CA-125 levels (ln-transformed)	0.213	0.077	0.006
Primary treatment strategy			
PDS	1		
NAC	1.039	0.220	<0.001
Pelvic LN status			
No metastasis	1		
Metastasis	0.823	0.228	<0.001
Involvement of pelvic tissue other than uterus and tube			
No	1		
Microscopic	0.444	0.451	0.324
Macroscopic	−0.166	0.256	0.517
Involvement of small bowel and mesentery			
No + Microscopic	1		
Macroscopic, ≤2 cm	0.812	0.231	<0.001
Macroscopic, >2 cm	0.672	0.404	0.097

Abbreviations: CA-125, cancer antigen 125; LN, lymph node; NAC, neoadjuvant chemotherapy; PDS, primary debulking surgery; SE, standard error.

© 2021 by the authors. Licensee MDPI, Basel, Switzerland. This article is an open access article distributed under the terms and conditions of the Creative Commons Attribution (CC BY) license (http://creativecommons.org/licenses/by/4.0/).
